# Persistent respiratory failure after SARS-CoV-2 infection: The role of dual energy computed tomography. A case report^☆^

**DOI:** 10.1016/j.radcr.2022.05.031

**Published:** 2022-06-28

**Authors:** Silvia Menale, Valentina Scheggi, Jacopo Giovacchini, Niccolò Marchionni

**Affiliations:** a Division of Cardiovascular and Perioperative Medicine, Cardiothoracovascular Department Azienda Ospedaliero-Universitaria Careggi and Department of Experimental and Clinical Medicine, University of Florence, Largo Brambilla 3, 50139, Italy; b Division of General Cardiology, Careggi Hospital and Department of Experimental and Clinical Medicine, University of Florence, Largo Brambilla 3, 50139, Italy

**Keywords:** Persistent respiratory failure, SARS-COV-2, Pulmonary microembolism, Dual energy CT (DECT), Case report, ACE, angiotensin converting enzyme, CT, computed tomography, CTPA, CT pulmonary angiography, DECT, dual-energy computed tomography, ED, emergency department, LMWH, low molecular weight heparin, PE, pulmonary embolism, PPS, pulmonary perfusion scintigraphy, TTE, trans-thoracic echocardiography, vWF, von Willebrand factor

## Abstract

*Background:* COVID-19 disease is often complicated by respiratory failure, developing through multiple pathophysiological mechanisms, with pulmonary embolism (PE) and microvascular thrombosis as key and frequent components. Newer imaging modalities such as dual-energy computed tomography (DECT) can represent a turning point in the diagnosis and follow-up of suspected PE during COVID-19. *Case presentation:* A 78-year-old female presented to our internal medicine 3 weeks after initial hospitalization for COVID-19 disease, for recrudescent respiratory failure needing oxygen therapy. A computed tomography (CT) lungs scan showed a typical SARSCoV-2 pneumonia. Over the following 15 days, respiratory function gradually improved. Unexpectedly, after 21 days from symptom onset, the patient started complaining of breath shortening with remarkable desaturation requiring high-flow oxygen ventilation. CT pulmonary angiography and transthoracic echocardiography were negative for signs of PE. Thereby, Dual-energy CT angiography of the lungs (DECT) was performed and detected diffuse peripheral microembolism. After 2 weeks, a second DECT was performed, showing a good response to the anticoagulation regimen, with reduced extent of microembolism and some of the remaining emboli partially recanalized. *Discussion:* DECT is an emerging diagnostic technique providing both functional and anatomical information. DECT has been reported to produce a much sharper delineation of perfusion defects than pulmonary scintigraphy, using a significantly lower equivalent dose of mSv. We highlight that DECT is particularly useful in SARS-Cov-2 infection, in order to determine the predominant underlying pathophysiology, particularly when respiratory failure prolongs despite improved lung parenchymal radiological findings

## Introduction

Progressive respiratory failure is a major cause of death in COVID-19 disease [1]. The underlying pathophysiologic mechanism leading to respiratory failure is multifactorial including, beyond inflammation, endothelial damage, intussusceptive angiogenesis, and microvascular thrombosis [1,2] On the other hand, the diagnosis of pulmonary embolism (PE) or thrombosis may be sometimes difficult, and the most used tools cannot always exclude this diagnostic suspicion, because microembolization or in situ thrombosis in the most peripheral regions of the lung are difficult to be detected. In front of such a challenging diagnostic scenario, dual-energy computed tomography (DECT), may result more informative and, hence, clinically helpful. In fact, DECT can produce a detailed definition of both morphology and perfusion in a single spiral image with secondary iodine maps using 2 X-ray energy spectra, high and low kilovoltage [3,4].

## Case presentation

We report here the case of a 78-year-old woman who developed an acute respiratory insufficiency 1 month after hospitalization for SARS-CoV-2-related pneumonia.

Her past medical history included arterial hypertension, chronic bronchitis, and chronic hepatitis C virus infection. Previous invasive procedures included appendectomy and right carpal tunnel surgery. She had presented to the emergency department because of increasing shortness of breath and fever up to 37.5°C in the last 5 days. The molecular swab test was positive for SARS-CoV-2. The patient was alert and oriented; the respiratory rate was slightly increased to 20/min. Arterial blood gases analysis revealed mild hypoxia with respiratory alkalosis (pH 7.46, pCO_2_ 37.7 mm Hg, pO_2_ 71 mm Hg, SPO_2_ 95% with FiO_2_ 21%). Chest ultrasound showed normal pleural sliding bilaterally, B-lines up to 6-7 on examination, and no pleural effusion. At chest radiography, disseminated areas of increased density in the lung parenchyma were detected. After initial triage, she was admitted to the division of infectious disease, where she was treated with intravenous steroids (methylprednisolone 60 mg die), LMWH at a prophylactic dose and oxygen therapy (FiO_2_ 36%-50%). A computed tomography (CT) lungs scan showed a relevant thickening of the parenchyma and bilateral ground-glass opacities, predominantly in peripheral and posterior regions, with overall typical aspects of SARS-CoV-2 pneumonia. Echocardiography demonstrated a preserved left ventricular systolic function and moderate aortic valve regurgitation, without signs of pulmonary hypertension.

Over the following 10 days, respiratory function gradually improved until almost complete weaning from O_2_ therapy. Unexpectedly, 21 days after symptom onset, the patient started complaining of breath shortening with relevant desaturation (SpO_2_ 87%), associated with chest tightness, requiring high-flow nasal cannula oxygen delivery (40 l/min with 60% FiO_2_).

**Fig. 1:**
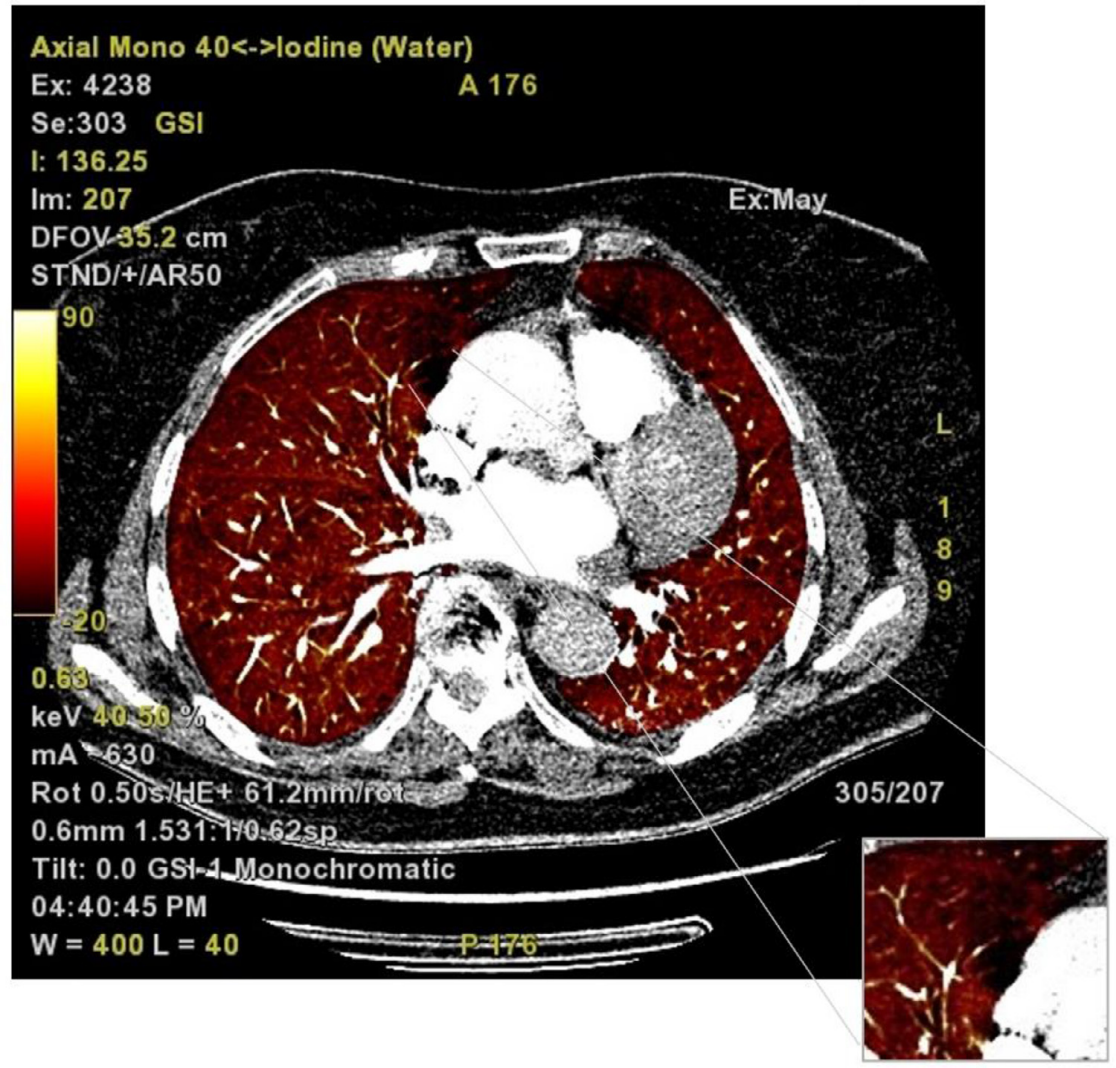
Iodine perfusion map-arterial phase. Focal-low perfusion zones due to subsegmental arterial small vessels embolism, localized at the lateral and medial segment of the medium right pulmonary lobe.

**Fig. 2:**
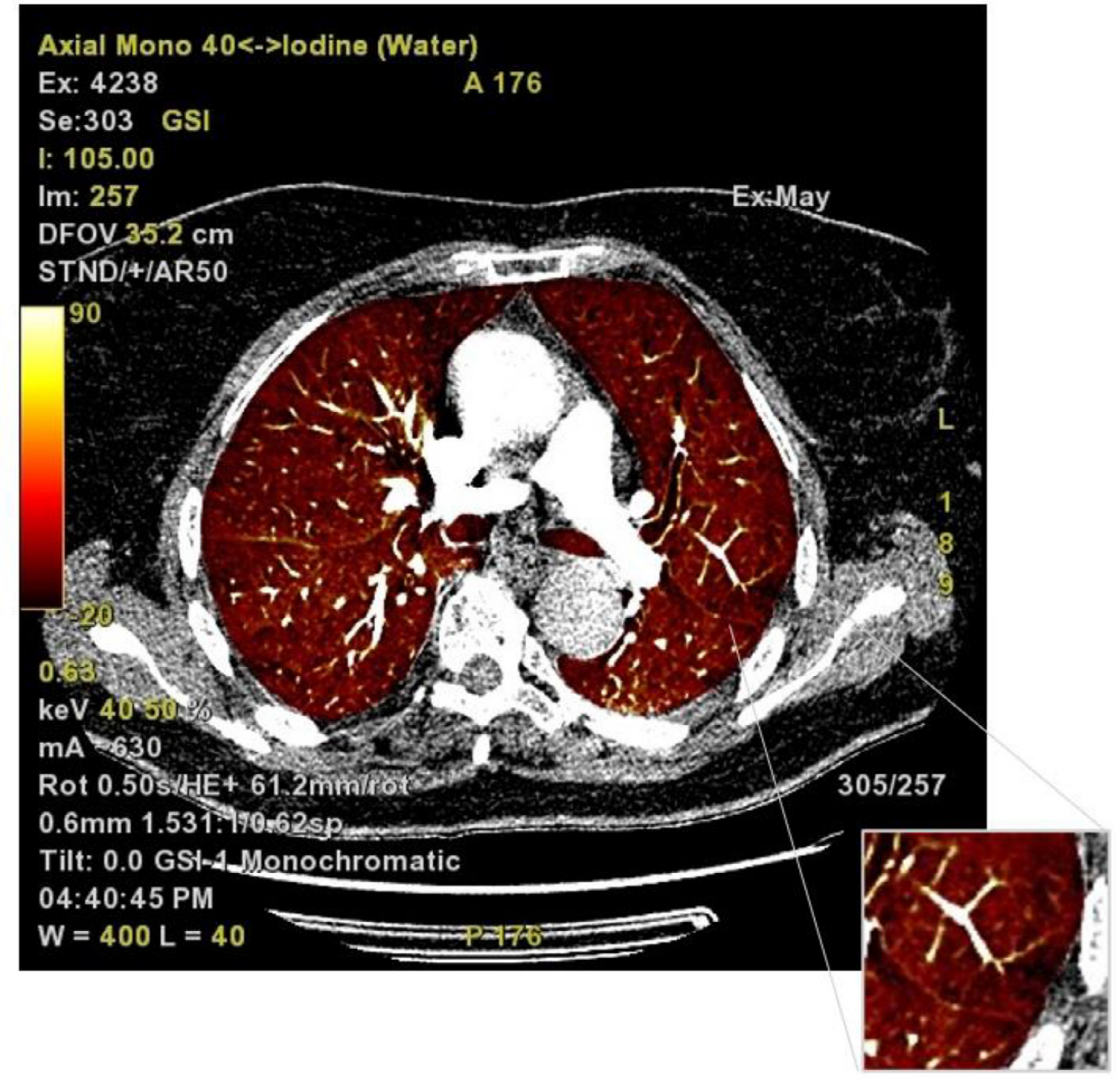
Iodine perfusion map-arterial phase. Focal-low perfusion zones with irregular and filiform arterial vessels, at lateral-basal segment of the inferior left pulmonary lobe.

A pulmonary CT angiography excluded a new-onset PE or progression of viral-related pneumonia. A subsequent DECT detected, at iodine perfusion maps, multiple triangular peripheral focal areas of hypoperfusion that were consistent with diffuse microembolism (Figs. 1 and 2).

LMWH therapy was then increased to an anticoagulant dosage. The respiratory profile remained stable with 50% FiO_2_. Meanwhile, the patient tested negative for SARS-CoV-2 at a control swab and was moved to our division of internal medicine.

**Fig. 3:**
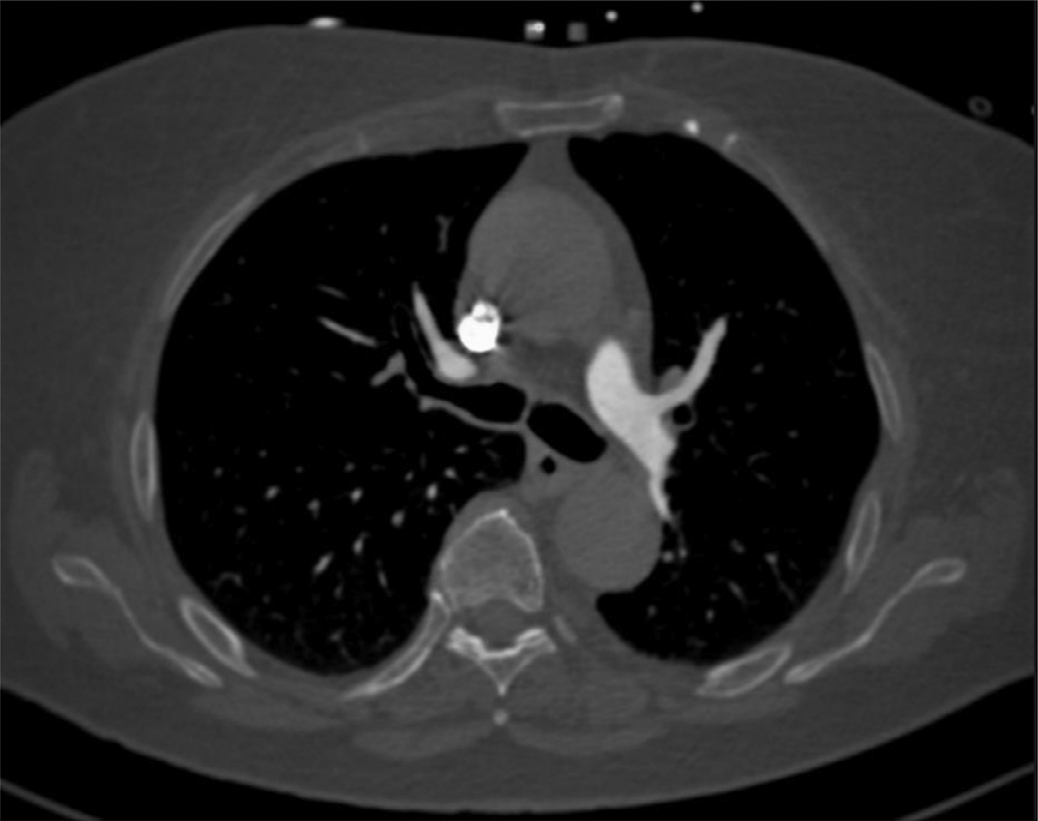
pulmonary arterial phase, Chest CT. Complete vessel recanalization, perfusion defects are no longer noticeable.

On admission, laboratory data showed normal haematocrit and inflammatory markers, with persistently high D-dimer levels (1217 ng/mL), while liver and cardiac enzymes, and renal function were normal. The patient still needed the same ventilatory support, with moderate hypoxemia and respiratory alkalosis at hemogasanalysis (pH 7.30, pO_2_ 57 mm Hg, pCO_2_ 31 mm Hg). After three weeks from admission to our division, a second DECT was performed, showing a good response to the anticoagulation regimen translating into a reduced extension of microemboli, with some of the remaining ones that were partially recanalized. A medium-dose corticosteroids and oxygen therapy were maintained, combined with respiratory physiotherapy and daily periods of pronation. The patient's general condition and respiratory function gradually improved, requiring only a small amount of oxygen support at discharge. A further, 3-month contrast-enhanced chest CT control, showed almost complete peripheral vessels recanalization and reduced areas of parenchymal consolidation (Fig. 3).

## Discussion

A series of specific events correlated with disease severity have been observed to contribute to the procoagulant state in COVID-19 disease: direct and indirect endothelial damage with activation of the coagulation pathway, platelet activation, complement and immune activation with the onset of an inflammatory cytokine storm involving IL2, IL6, MCP-1, MIP-1A, and TNF-α [5,6]. This process, termed immunothrombosis, complements other additional risk factors (high ferritin, elevated antiphospholipid antibodies, obesity) and the classic venous thromboembolism pathways that characterise COVID-19 [2,6].

Moreover, inflammation-induced endothelial cell dysfunction leads to an increased release of von Willebrand factor (vWF) multimers, triggering thrombotic microangiopathy [7]. ADAMTS-13 levels and ultra-large vWF multimers in patients with sepsis is inversely correlated, as several studies asserted. More recently, Bazzan et al. reported significantly reduced ADAMTS-13 levels in a cohort of COVID-19 patients. They found that ADAMTS-13 levels below 30% of the normal values, combined with high plasma vWF levels, predicted an increased risk of death in COVID-19 [8].

Thrombotic events occur in more than 30% of patients with COVID-19 predominantly affecting the lungs, and are associated with more severe disease and higher mortality [1,5]. It is well known how SARS-CoV-2 favors the respiratory tract, where its entrance into target cells occurs through an angiotensin-converting enzyme 2 receptor, as well as in other organs such as the kidneys, heart, brain, and liver [2,^5^,6].

While PE has been detected in around 5%-14% of COVID-19 patients [9, 10], it is important to notice that such reported prevalence likely underestimated the real numbers of COVID-19-related PE, since CT pulmonary angiography (CTPA) has not been performed in all cases.

Indeed, in case of suspected PE, conventional CTPA is the first-line recommended diagnostic method. However, as highlighted by the clinical case we have described, its sensitivity in assessing the involvement of small-diameter (ie, subsegmental) pulmonary arteries is variable, and provides anatomical information only. An optimal definition of pulmonary artery subsegmental branches has been developed with the newest CT techniques, such as the combination with iodine map in the DECT method, providing both morphological and detailed functional information [11].

Therefore, when a PE of subsegmental segments is suspected because of prolonged respiratory distress despite reduced parenchymal inflammation, further imaging techniques are needed, with lung ventilation/perfusion scintigraphy or DECT as possible alternatives, with some difference.

DECT displays pulmonary perfusion defects in good agreement with perfusion scintigraphy [12], but provides more detailed high-quality images of the anatomy of pulmonary circulation. Indeed, analysis from the same datasets demonstrated to what extension DECT was more accurate than perfusion scintigraphy in the detection of PE [13,14], and DECT provides a remarkably sharper delineation of perfusion defects with lower equivalent radiation [15].

Based on this evidence, our patient underwent first a conventional CTPA but then, because of the persistent respiratory failure, was further studied with DECT, in order to more accurately search for PE. In our clinical case, the choice to perform DECT rather than pulmonary scintigraphy was also related to a simpler and faster access to this diagnostic method, which is also well reproducible in the follow-up [15].

DECT is an emerging imaging technique based on the acquisition of data using 2 X-ray energy spectra, high and low kilovoltage, within a single scan. In fact, if conventional single-energy examination CT produces only a single set of images, dual-energy data can be used to reconstruct numerous types of images, such as weighted average images, material decomposition images, and electron density mapping that allows structures with similar density to be distinguished according to their electron density differences [4].

Employment of an iodine-containing contrast agent improves the signal to noise ratio, and allows quantitative measurement of the contrast agent in the lungs through the generation of iodine maps as an indicator of pulmonary blood flow [16]. Tissue discrimination is based on differential attenuation of the radiation beam. Retro-reconstruction is then used to create a distribution map of the iodinated contrast agent, which allows us to examine and highlight conditions of the pulmonary circulation, even at the parenchymal level [16].

Iodine maps increase the specificity of PE detection as well, enabling a quantitative analysis of the volume of perfusion defect, thereby reducing false positivity and overdiagnosis [17]. It provides a pulmonary CT angiogram with high spatial resolution of small vessels and parenchyma at the same time, with no additional radiation exposure [12].

However, false-negative or false-positive results of DECT for perfusion or ventilation defects due to motion artefacts or excessive contrast must be considered as possible methodological limitations. Moreover, DECT its use is limited in morbidly obese patients because of the interference with structural and functional image analysis [18].

## Conclusions

In summary, despite some abovementioned limitations, DECT can provide superior both anatomic and functional information in various lung diseases [19]. This tool is particularly useful in COVID-19 with marked or prolonged respiratory distress after first-line radiological thoracic examination, in order to determine the predominant underlying pathophysiology and, hence, individually tailor the therapeutic approach.

## Ethics approval and consent to participate

Not applicable.

## Patient consent

Written informed consent was obtained from the patient.

## Availability of data and materials

Not applicable.

## References

[1] Ackermann M, Verleden SE, Kuehnel M, Haverich A, Welte T, Laenger F (2020). Pulmonary vascular endothelialitis, thrombosis, and angiogenesis in Covid-19. New Engl J Med.

[2] Henry BM, Vikse J, Benoit S, Favaloro EJ, Lippi G. (2020). Hyperinflammation and derangement of renin-angiotensin-aldosterone system in COVID-19: a novel hypothesis for clinically suspected hypercoagulopathy and microvascular immunothrombosis. Clin Chim Acta.

[3] Furlow B. (2015). Dual-energy computed tomography. Radiol Technol.

[4] Baerends E, Smit CT, Das M, Sechopoulos I, Brink M, Oostveen LJ, Smit CT (2018). Comparing dual energy CT and subtraction CT on a phantom: which one provides the best contrast in iodine maps for sub-centimetre details?. Eur Radiol.

[5] Loo J, Spittle DA, Newnham M. (2021). COVID-19, immunothrombosis and venous thromboembolism: biological mechanisms. Thorax.

[6] Gheblawi M, Wang K, Viveiros A, Nguyen Q, Zhong JC, Turner AJ (2020). Angiotensin-converting enzyme 2: SARS-CoV-2 receptor and regulator of the renin-angiotensin system: celebrating the 20th anniversary of the discovery of ACE2. Circ Res.

[7] Levi M, Scully M, Singer M. (2018). The role of ADAMTS-13 in the coagulopathy of sepsis. Thromb Haemost.

[8] Bazzan M, Montaruli B, Sciascia S, Cosseddu D, Norbiato C, Roccatello D (2020). Low ADAMTS 13 plasma levels are predictors of mortality in COVID-19 patients. Intern Emerg Med.

[9] Miró O, S Jiménez, Mebazaa A, Freund J, Burillo-Putze G, Martìn A (2021). Pulmonary embolism in patients with COVID-19: incidence, risk factors, clinical characteristics, and outcome. Eur Heart J.

[10] Jevnikar M, Sanchez O, Chocron R, Andronikof M, Raphael M, Meyrignac O (2021). Prevalence of pulmonary embolism in patients with COVID 19 at the time of hospital admission. Eur Respir J.

[11] Lu GM, Wu SY, Yeh BM, Zhang LJ. (2010). Dual-energy computed tomography in pulmonary embolism. Br J Radiol.

[12] Thieme SF, Becker CR, Hacker M, Nikolaou K, Reiser MF, Johnson TR. (2008). Dual-energy CT for the assessment of lung perfusion–correlation to scintigraphy. Eur J Radiol.

[13] Aran S, Besheli LD, Karcaaltincaba M, Gupta R, Abujudeh HH, Flores EJ (2014). Applications of dual-energy CT in emergency radiology. AJR.

[14] Masy M, Giordano J, Petyt G., Hossein-Foucher C, Kyheng M, lain Duhamel A (2018). Dual-energy CT (DECT) lung perfusion in pulmonary hypertension: concordance rate with V/Q scintigraphy in diagnosing chronic thromboembolic pulmonary hypertension (CTEPH). Eur Radiol.

[15] Si-Mohamedab S, Moreau-Tribyc C, Tylskid P, Tatard-Leitman V, Wdowik Q, Boccalini S (2020). Head-to-head comparison of lung perfusion with dual-energy CT and SPECT-CT. Diagn Interv Imaging.

[16] McCollough CH, Leng S, Yu L, Fletcher JG. (2015). Dual- and multi-energy CT: principles, technical approaches, and clinical applications. Radiology.

[17] Grob D, Smit E, Prince J, Kist J, Kist J, Stöger L (2019). Iodine maps from subtraction CT or dual-energy CT to detect pulmonary emboli with CT angiography: a multiple-observer study. Radiology.

[18] Masy M, Giordano J, Petyt G, Hossein-Foucher C, Duhamel A, Kyheng M (2018). Dual-energy CT (DECT) lung perfusion in pulmonary hypertension: concordance rate with V/Q scintigraphy in diagnosing chronic thromboembolic pulmonary hypertension (CTEPH). Eur Radiol.

[19] Capone C, Valentini A, Spinillo SL, Klersy C, Klersy C, Celentano A (2021). Radiological differences between chronic thromboembolic pulmonary disease (CTEPD) and chronic thromboembolic pulmonary hypertension (CTEPH). Eur Radiol.

